# Lipoprotein Lipase HindIII Intronic Polymorphism in a Subset of Iranian Patients with Late-Onset Alzheimer’s Disease

**Published:** 2012-06-13

**Authors:** Azadeh Sayad, Mehrdad Noruzinia, Mahdi Zamani, Mohammad Hossein Harirchian, Anoshirvan Kazemnejad

**Affiliations:** 1. Department of Medical Genetics, School of Medical Sciences, Tarbiat Modares University, Tehran, Iran; 2. Department of Neurogenetics, Iranian Centre of Neurological Research, Tehran University of Medical Sciences, Tehran, Iran; 3. Department of Biostatistics, Tarbiat Modares University, Tehran, Iran

**Keywords:** Alzheimer’s Disease, LPL Gene, HindIII Polymorphism, Association Study

## Abstract

**Objective:**

Lipid metabolism is involved in the pathogenesis of late-onset Alzheimer’s disease (LOAD). Lipoprotein lipase (LPL) is a multifunctional enzyme that plays a major role in lipid metabolism; its abnormal function seems to be related, either directly or indirectly, to the pathogenesis of many diseases such as atherosclerosis, coronary artery disease (CAD) and Alzheimer’s disease (AD) . HindIII polymorphism is a common LPL genetic variant shown to increase the risk of LOAD. The present research investigates whether this polymorphism is involved in the pathogenesis of Iranian LOAD patients.

**Materials and Methods:**

In this case control study ,allele and genotype frequencies for the HindIII polymorphism of the LPL gene in 100 patients affected with LOAD and 100 healthy controls were determined by reaction-restriction fragment length polymorphism (PCR-RFLP) and compared using the chi-square and Fisher’s exact tests.

**Results:**

LPL H+H+ genotype frequency in LOAD patients was 58%, which was significantly higher than controls (44%). There was a 1.75-fold increased risk for the development of LOAD in carriers of the H+H+ genotype compared to non-carriers (OR=1.75; 95%CI: 1.00-3.07; p=0.048). When adjusted for sex, the H+H+ genotype was more frequent in patients than controls; this difference was more remarkable in males (OR: 1.90; 95% CI: 1.08-3.34; p=0.024). The mean age of disease onset did not differ in patients with the LPL H+H+ genotype compared to unaffected individuals.

**Conclusion:**

This study confirms the association between the H+H+ genotype with LOAD and supports the correlation of this genotype of the LPL gene with risk of developing LOAD in Iranian patients with AD.

## Introduction

Alzheimer’s disease (AD) is the most prevalent cause of dementia in the elderly with a genetically complex pattern. AD, based upon the age at onset, either before or after about 60 years, is categorized into two types of early and late onset.

The majority of Alzheimer’s patients are diagnosed with the late-onset form of the disease, most of which are non-familial. Complex inheritance and multi-factorial patterns of late-onset Alzheimer’s disease (LOAD) along with its heterogeneity are basically due to the presence of different predisposing genes and intra-ethnic variability.

Meanwhile, a series of molecular studies on LOAD in some countries have shown that in addition to apolipoprotein E (ApoE), the only known gene associated with LOAD whose gene product functions as a common transporter of lipid in the brain, there are other important, population-dependent genes associated with this disease.

 Senile plaques and neurofibrillary tangles are the hallmarks of the diagnosis of AD. In AD, amyloid-β protein (Aβ) aggregates in the brain to form amyloid plaques, which also contain many other proteins such as ApoE, low-density lipoprotein (LDL) receptor-related protein (LRP) and LPL (1).

Lipoprotein lipase (LPL) is an enzyme involved in the hydrolysis of triglycerides and very-low density lipoproteins, in addition to the production of free fatty acids and monoglycerides. LPL is found in many organs including the brain and it has a three-fold higher activity in the hippocampus compared to other areas of the brain (2,3). LPL transports cholesterol and vitamin E to neurons. Recently, in addition to hydrolysis, LPL has been reported to be involved in lipid intake and clearance (2-5). Together these findings suggest a role for LPL in regulation of cognitive function and leads to the notion that polymorphisms in the LPL gene might modify the risk of AD.

The LPL gene is located on chromosome 8p22, spanning approximately 35 kilobases. It contains 10 exons and encodes a 448-amino acid mature protein (6). In addition to major gene rearrangements and missense mutations, which cluster in exons 4, 5, and 6 several restriction fragment length polymorphisms (RFLPs) have been reported at the LPL locus(7-13), among which *Hind*III in intron 8 (T→G at position 481) is of particular interest because of its common occurrence (25% of the less common alleles in most populations) and association with plasma lipid profile (2-5, 7). as well as susceptibility to coronary artery disease (CAD) (2,8-12) in several studies. However although some inconsistent results have also been reported (13-15).

Since the *Hind*III polymorphism is located in the middle of intron 8, it is not considered to be functional but rather in linkage disequilibrium with a putative functional variant. It may exert a possible action on other parts of the gene, or have a direct functional role by itself (11,16).

To our knowledge only one preliminary study has investigated the distribution of intronic HindIII polymorphisms in AD, and involved 243 Italian AD patients. The researchers reported a significantly increased risk for H+ allele in cases (15).

While a number of LOAD genetic risk factors have been studied in Iranian patients, the role of the HindIII polymorphism in this population has not been clarified. Therefore the current study seeks to investigate the prevalence of *Hind*III polymorphism in a group of Iranian patients with LOAD in comparison with normal controls.

## Materials and Methods

In this study, 100 Iranian patients affected with AD and 100 normal controls, who had no family history of AD, were studied. The age of onset for patients was 76.44 ± 5.60 years. Cases and controls were matched by sex, age, ethnic background, and geographic location. Our patients were gathered from the Dementia Outpatient Clinic of the Iranian Alzheimer Association (IAA). All patients or their legal guardians and all controls signed written informed consents. This study was approved by the Medical Ethics Committee at Tarbiat Modares University. Blood samples were collected from 50 men and 50 women with AD. Patients were confirmed to have AD based on clinical examination by two experienced neurologists at Imam Khomeini Hospital, Tehran, Iran and IAA according to Diagnostic and Statistical Manual of Mental Disorders, Fourth edition, Text Revision ( DSM-IV-TR) (17) and National Institute of Neurological and Communicative Diseases and Stroke/Alzheimer’s Disease and Related Disorders Association (NINCDS-ADRDA) (18) clinical diagnostic criteria. These diagnostic criteria are based upon neurological examinations, neuropsychological tests, and brain imaging studies. The control group comprised 100 apparently healthy unrelated subjects over the age of 65 years who had no neurodegenerative disorders and no history of either AD or dementia. Cognitive function was assessed using the Mini-Mental State Examination (MMSE) (19). Subjects who scored greater than 28 were included.

DNA was extracted from peripheral blood lymphocytes using the salting out procedure (20). The method used to detect LPL intronic HindIII polymorphism was based on polymerase chain reaction-restriction fragment length polymorphism (PCR-RFLP). Amplification reaction for the studied polymorphism contained 500 ng DNA (10 pmol) of each primer, 1.5 u of Taq DNA polymerase, 200 mM dNTP (0.5 µl), and 2.5 mM MgCl.

**Fig 1:**
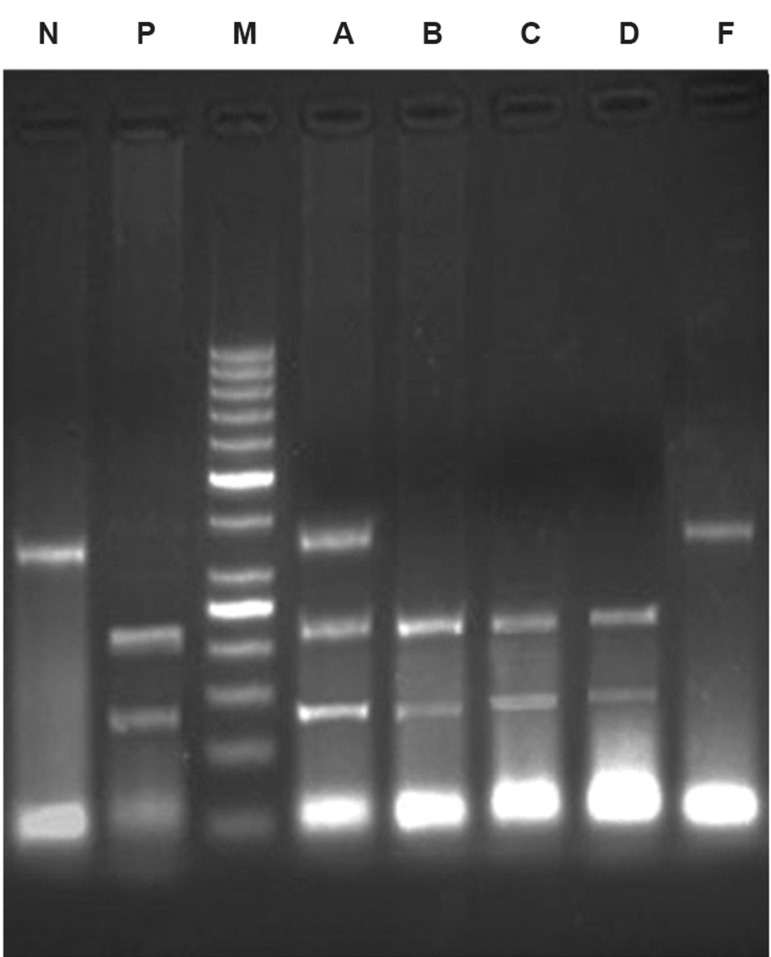
Electrophoresis pattern of HindIII polymorphism. M: Marker (50 bp); N: No digestion (350 bp); P: Positive control (140 and 210 bp); B, C, D: H+H+ genotype (140 and 210 bp); A: H+H- genotype (140, 210, and 350 bp); F: H-H- genotype (350 bp).

**Fig 2:**
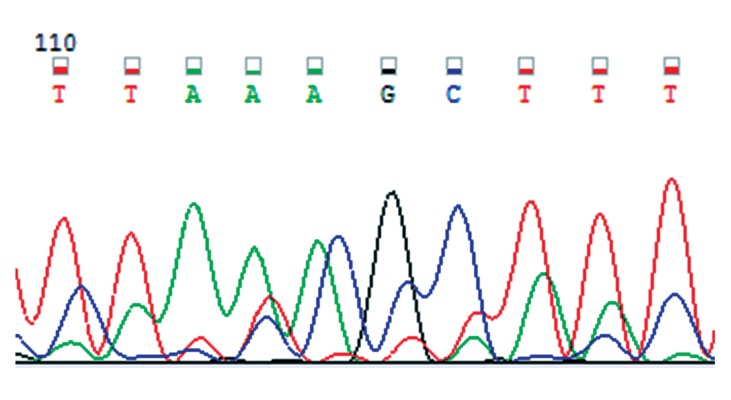
DNA sequence of H+H+ genotype sample

The primer set for LPL (18) was: 5´-GATGTCTACCTGGATAATCAAAG-3´(LPLF) and 5´-CTTCAGCTAGACATTGCTAGTGT-3´ (LPLR). The reaction mixture after denaturation for 5 minutes at 94℃ was subjected to 35 cycles of 0.5 minutes at 94℃, 0.5 minutes at 59℃, and 1.5 minutes at 72℃ followed by a final extension period of 5 minutes at 72℃. The PCR product was digested overnight by HindIII enzyme and visualized on 1.5% agarose gel. The polymorphism yielded fragments of 210 and 140 bps after digestion (Fig 1). To confirm the results of PCR-RFLP, direct sequencing was performed on a DNA sample with H+/H+ genotype using an ABI 3031XL automatic sequencer (Fig 2).

### Statistical analysis

Comparisons of groups were performed using the chi-square and Fisher’s exact tests. Odds ratio, 95% confidence intervals (CI), and p values were also calculated to further estimate the association between the polymorphism and the developing risk of AD.

## Results

The data for genotype and allele distribution of HindIII polymorphisms, odds ratio and p value of the association between the HindIII polymorphism and the risk for developing AD among study participants are presented in table 1.

Analysis of our data demonstrated a significant overrepresentation of the H+H+ genotype of the LPL gene in AD patients (58%) compared to healthy subjects (44%). The presence of the H+H+ genotype led to a 1.75-fold increase in the risk for developing LOAD compared to carriers of H-H-and H+H- genotypes (OR=1.75; 95% CI: 1.00 – 3.07; p=0.048; table 1). However, carriers of the H+ allele as noted in 78% of AD patients compared to 70% of controls, gave an OR of 1.51 for developing the disease (OR=1.51; 95% CI: 0.98–2.38; p=0.068).

When the association between LPL genotypes and AD was categorized by gender, LPL H+H+ genotype frequency in patients was higher than controls in both genders. This differentiation was more remarkable in males than females with an estimated OR of 1.9 (95% CI: 1.08 – 3.34; p=0.024), but was not significant in females (p=0.090, Table 1). The mean age at onset of LOAD was not significantly different in patients with H+H+ genotype (75.87 ± 5.90) compared to patients with the H+H- genotype (77.15 ± 5.25).

**Table 1:** Genotype and allele distribution of studied polymorphism in healthy controls and AD patients


	Polymorphism	Genotype frequency(%)			OR	95%C	Allele frequency(%)			OR	95%C	
	Hind III	H+H+	H+H-	H- H-		*I	P	H+	H-		I	P

Total	CTR	44	52	4	1.7	1.00 -	0.0	70	30	1.5	0.96 -	0.0
	AD	58	40	58	5	3.07	48	78	22	1	2.38	68
M	CTR	44	50	44	1.9	1.08 -	0.0	69	31	1.5	1.07 -	0.0
	AD	60	36	60	0	3.34	24	78	22	9	2.49	41
F	CTR	44	54	44	1.6	0.92-	0.0	71	29	1.4	0.92-	0.1
	AD	56	44	56	2	2.83	90	78	22	4	2.27	08


CTR: Controls, AD: Patients with Alzheimer's disease, M: Males, F: Females. *H+H+ vs. H+H-/H-H-

## Discussion

In this study we determined that the LPL HindIII intronic polymorphism conferred an increased risk of LOAD among Iranians. Baun et al. in 1999 has shown that LPL was involved in the pathophysiology of AD. However the authors found that different polymorphisms of this gene could have a putative causative or protective effect for AD (5). Evidence has supported a relationship between vascular risk factors and AD. Stroke, diabetes, hypertension, smoking, heart disease, dyslipidemia, and obesity have been implicated in the risk of acquiring LOAD (21). In another recent longitudinal SPECT study it has been shown that multiple vascular risk factors are associated with a greater rate of decline of mental functions in patients with AD (22). However, studies have shown that ethnicity and age, as well as numerous additional factors possibly influence the risk of genetic polymorphisms in AD development. Myllykangashas shown in a Finnish study of 113 elderly patients with clinically defined AD (NINCDS-ADRDA criteria) and 203 normal controls, that LPL and LRP polymorphisms had a slight impact on the risk of AD (23).

Studies of the LPL HindIII intronic polymorphism and its effect on the development of AD have shown that in some populations there is an increased risk of LOAD. Blain et al. have shown in a post mortem group of eastern Canadian patients with confirmed AD that a common polymorphism in the LPL gene modulated the risk level for sporadic AD. These researchers have shown more importantly that this polymorphism indirectly modulated the brain's pathophysiology(24).

In the current study, we determined that the H+ allele did not alter the risk of AD as the p values were not significant. Our results did not confirm an Italian study on patients with AD, in which Scacchi et al. reported in a group of 243 Italian patients with sporadic LOAD that the odds ratio from the logistic regression analysis for the H+H+ genotype was 1.39 (95% CI:0.92–2.1; p=0.15).

However in this study, the H+ allele difference between controls and cases was not significant (p=0.059) (15). In a larger population of patients with LOAD in Switzerland, Papassotiropoulos et al. have shown that a cluster of polymorphisms in APOE, SOAT1, the APOE 5'-untranslated region, OLR1, CYP46A1, LPL, LIPA, and APOA4 conferred significant (p=0.0002) susceptibility for AD (25), which was confirmed by the current study which showed that patients with LPL polymorphism in the homozygous state had a greater risk for developing LOAD.

HindIII polymorphism is located in the middle of intron 8 (T→G at position 481) and does not cause an amino acid change in the protein. However, the association of this polymorphism with CAD, hypertriglyceridemia, and hypertension (7, 8) has been shown in many studies.

The mechanism is unknown but a linkage disequilibrium with a functional mutation arising in another part of the gene (9) is not excluded. A direct functional role has been proposed by other researchers (26).

 Many researchers are interested in developing a vascular risk score to identify elderly individuals who might be at risk for LOAD (27). This risk score could also be used to adjust for confounders in epidemiologic studies. Our study shows that in the Iranian population, the LPL HindIII intronic polymorphism can be considered as a candidate in a similar risk calculation scoring system. These findings can help us to define a more specific nationwide risk calculation of LOAD. However, our study limitation is its sample size. Further studies utilizing larger numbers of subjects are necessary to evaluate additional AD risk factors in Iran.

## Conclusion

This is the first study to report the role of the LPL HindIII intronic polymorphism in Iranian patients with LOAD. Our results could assist in the development of an ethnic-specific risk assessment scoring system in the Iranian population. In addition, these results can confirm the role of lipid metabolism modifiers in the pathogenesis of LOAD in Iran. On the other hand, this study has shown that the H+H+ genotype is a risk factor, yet we could not confirm the role of the H+ allele in the pathogenesis of LOAD. Further studies are needed to evaluate additional risk factors of LOAD in the Iranian population.
